# Complete Genome Sequence of the First Isolate of Genotype C Bovine Parainfluenza Virus Type 3 in Japan

**DOI:** 10.1128/genomeA.01215-14

**Published:** 2014-11-26

**Authors:** Misako Konishi, Takashi Ohkura, Madoka Shimizu, Masanori Akiyama, Ken-ichiro Kameyama, Kaoru Takeuchi

**Affiliations:** a Viral Diseases and Epidemiology Research Division, National Institute of Animal Health, Tsukuba, Ibaraki, Japan; b Department of Infection Biology, Division of Biomedical Science, Faculty of Medicine, University of Tsukuba, Tsukuba, Ibaraki, Japan; c Western Center for Livestock Hygiene Service, Higashihiroshima, Hiroshima Prefecture, Hiroshima, Japan

## Abstract

Bovine parainfluenza virus type 3 (BPIV3) isolates are classified into three genotypes (BPIV3a to -c). Here, we report the complete genome sequence of the BPIV3c isolate for the first time in Japan. Our results indicate that new primer sets will be required to detect all genotypes of BPIV3 strains.

## GENOME ANNOUNCEMENT

Bovine parainfluenza virus type 3 (BPIV3) is an enveloped, nonsegmented negative-sense RNA virus within the genus *Respirovirus* of the *Paramyxoviridae* family. BPIV3 causes severe respiratory illness in cattle, associated with bovine respiratory disease complex worldwide. Recent phylogenetic analyses revealed that BPIV3 viruses can be classified as genotypes A, B, and C (1–4). According to this classification, all Japanese strains published to date have been genotype A (BPIV3a) (1, 2, 5). To enable the broad detection of BPIV3 viruses in Japan, primer sets were designed based on the P gene of the 910N strain, a representative strain isolated in Japan, and subjected to reverse transcription (RT)-PCR (6). However, this primer set provided poor amplification of the RT-PCR product from viral RNA of BPIV3 isolate HS9, which was isolated from the nasal swab of a cow with respiratory symptoms in 2012. Direct PCR sequencing revealed that the isolate was closely related to the SD0835 strain, classified as genotype C (BPIV3c) (2). To determine the complete genome sequence of HS9, the isolate was propagated in Madin-Darby bovine kidney cells, and total RNAs were extracted from the infected cells. Overlapping cDNA fragments spanning the entire length of the genome were synthesized by RT-PCR using eight primer sets designed based on the sequence of the SD0835 strain. To determine the trailer sequence of the viral RNA genome, a 5′ full rapid amplification of cDNA ends (RACE) core set (TaKaRa) was used according to the manufacturer’s instructions. Subsequently, the cDNA of the leader sequence, which is complementary to the trailer sequence (7), was obtained using RACE or the primers designed based on the trailer sequence. Nucleotide sequences of cDNA fragments were determined using an ABI Prism 3130 Genetic Analyzer (Life technologies). The complete genome size of the isolate was 15,474 bp, which was identical to that of the SD0835 strain and six nucleotides shorter than that of the 910 N strain (2, 8, 9). Similarly to the SD0835 and 12Q061 strains (4), insertion of 12 nucleotides was detected in the P-gene (nucleotides 2504 to 2515 of the HS9 genome). The levels of identity of the complete genome sequences between the HS9 and the SD085 strains and between HS9 and the 12Q061 strains were 98.0% and 98.5%, respectively. Phylogenetic analysis indicated that the isolate HS9 was classified into genotype C. Genome organization was typical of BPIV3, with six genes (3′-N-P-M-F–HN-L-5′). Compared with the complete genome sequence of the 910N strain, the levels of sequence identity for each gene were 82.2% (N), 80.0% (P), 84.8% (M), 81.8% (F), 80.3% (HN), and 84.8% (L), respectively. To our knowledge, this is the first report of the complete genome sequence of the BPIV3c isolate in Japan. The low level of identity in the P-gene sequence between the HS9 isolate and 910N strain suggests that the current RT-PCR method using BPIV3a-specific primers will fail to detect BPIV3c strains. Avoiding the misdiagnosis of BPIV3 infection will require further sequence analysis of BPIV3 strains and the design of appropriate primers able detect all of its genotypes.

### Nucleotide sequence accession number.

The genome sequence of HS9 has been submitted to GenBank (accession number LC000638).
